# Integrative Long‐Read Multi‐Omics of a Patient With GPI Deficiency: A Molecular Case Study of a Candidate Dual‐Effect GPI Variant

**DOI:** 10.1111/jcmm.71338

**Published:** 2026-09-11

**Authors:** Ireneusz Stolarek, Joanna Delimata‐Raczek, Natalia Koralewska, Klaudia Sikora, Magdalena Rakoczy, Małgorzata Marcinkowska‐Swojak, Luiza Handschuh, Jarosław Czyż, Marek Figlerowicz

**Affiliations:** ^1^ Institute of Bioorganic Chemistry Polish Academy of Sciences Poznań Poland; ^2^ Ludwik Rydygier Collegium Medicum in Bydgoszcz Nicolaus Copernicus University in Toruń Bydgoszcz Poland; ^3^ Faculty of Medicine Bydgoszcz University of Science and Technology Bydgoszcz Poland

**Keywords:** allele specific expression, erythrocyte, GPI deficiency, integrative biology, multi‐omics

## Abstract

The molecular determinants of phenotypic severity in red cell enzymopathies are often obscured by the disconnect between coding sequence variants and their regulatory landscapes. Here we present a single‐patient molecular case study that uses an integrative multi‐omic approach—combining short‐read WGS, PacBio HiFi long‐read sequencing, native CpG methylation profiling, and Iso‐Seq full‐length transcriptomics—to characterize a severe, transfusion‐dependent hemolytic anaemia. We identified a compound heterozygous state in the glucose‐6‐phosphate isomerase (GPI) gene, with no wild‐type allele present. One allele (Haplotype 1) carried a missense variant (p.His191Arg); the other (Haplotype 2) carried a distinct missense variant, c.1414C>T (p.Arg472Cys), previously reported as biochemically unstable. Long‐read phasing placed the two variants in trans. Allele‐resolved transcript counts showed a directionally consistent but statistically non‐significant trend toward higher expression of Haplotype 2 across two Iso‐Seq replicates. Notably, the c.1414C>T transition abolishes a local CpG dinucleotide; in a small number of haplotype‐2 reads spanning this position, the corresponding cytosine on the wild‐type/Haplotype‐1 background was methylated. We did not measure GPI protein abundance, enzymatic activity, or stability in this patient, and we do not establish that methylation at this site regulates GPI transcription. On the basis of these correlative observations in a single patient, we propose—as a hypothesis for future testing—that a coding variant might simultaneously perturb protein stability and disrupt a local epigenetic mark, and we outline the experiments required to test whether such a dual effect contributes to disease. This case illustrates the value of integrative long‐read multi‐omics for generating mechanistic hypotheses about variants of uncertain significance, while underscoring that causal claims require dedicated functional validation.

## 1 Introduction

Glucose‐6‐phosphate isomerase (GPI) serves as a metabolic linchpin in glycolysis, catalysing the interconversion of glucose‐6‐phosphate and fructose‐6‐phosphate. While this process is ubiquitous, red blood cells (RBCs) are uniquely vulnerable to GPI dysfunction because they rely exclusively on glycolysis for ATP production and, crucially, lack the nuclear machinery to replenish enzymes once they mature [1]. Consequently, GPI deficiency (OMIM: 172400) typically manifests as hereditary nonspherocytic hemolytic anaemia (HNSHA), presenting a clinical spectrum that ranges from mild hemolysis to hydrops fetalis and neurological impairment [2, 3, 4].

Despite the characterization of over 40 pathogenic variants, the molecular determinants of clinical severity in GPI deficiency—and red cell enzymopathies in general—remain incompletely understood [5]. Traditional diagnostic workflows prioritize the identification of coding sequence mutations, largely under the assumption that protein structural defects are the principal drivers of pathogenicity. This “mutation‐centric” view can fail to explain apparent discordance between genotype and phenotype, where biallelic mutations occasionally result in milder disease than monoallelic variants, or where similar genotypes are associated with different clinical trajectories. Such discordance has led several groups to hypothesize that additional layers of regulation—including allele‐specific expression (ASE), cis‐regulatory elements, and epigenetic modifications—may contribute to disease severity, although direct evidence in red cell enzymopathies remains limited.

Standard short‐read sequencing has recognized limitations for resolving these regulatory features: short reads often cannot phase variants across long haplotypes or simultaneously capture native methylation, obscuring any interplay between transcriptional output and protein stability. To address this in a single, clinically severe case, we applied an integrative multi‐omics approach, leveraging PacBio HiFi long‐read genome sequencing and full‐length Iso‐Seq transcriptomics to characterize a severe, transfusion‐dependent phenotype.

Here we report a detailed molecular characterization of compound heterozygous variants in GPI in one patient. By integrating genomic, epigenomic, and transcriptomic data, we describe a candidate “dual‐effect” variant: a single nucleotide change that alters the coding sequence and also disrupts a local CpG dinucleotide. We use these observations to generate—rather than to prove—a testable hypothesis that such a variant might exert both structural and cis‐regulatory effects. Because the data are correlative and derive from a single patient, we frame these findings as hypothesis‐generating and outline the functional experiments needed to test causality.

## 2 Results

### 2.1 Phasing of Long‐Read WGS Data Resolves Compound Heterozygosity in Trans

**FIGURE 1:**
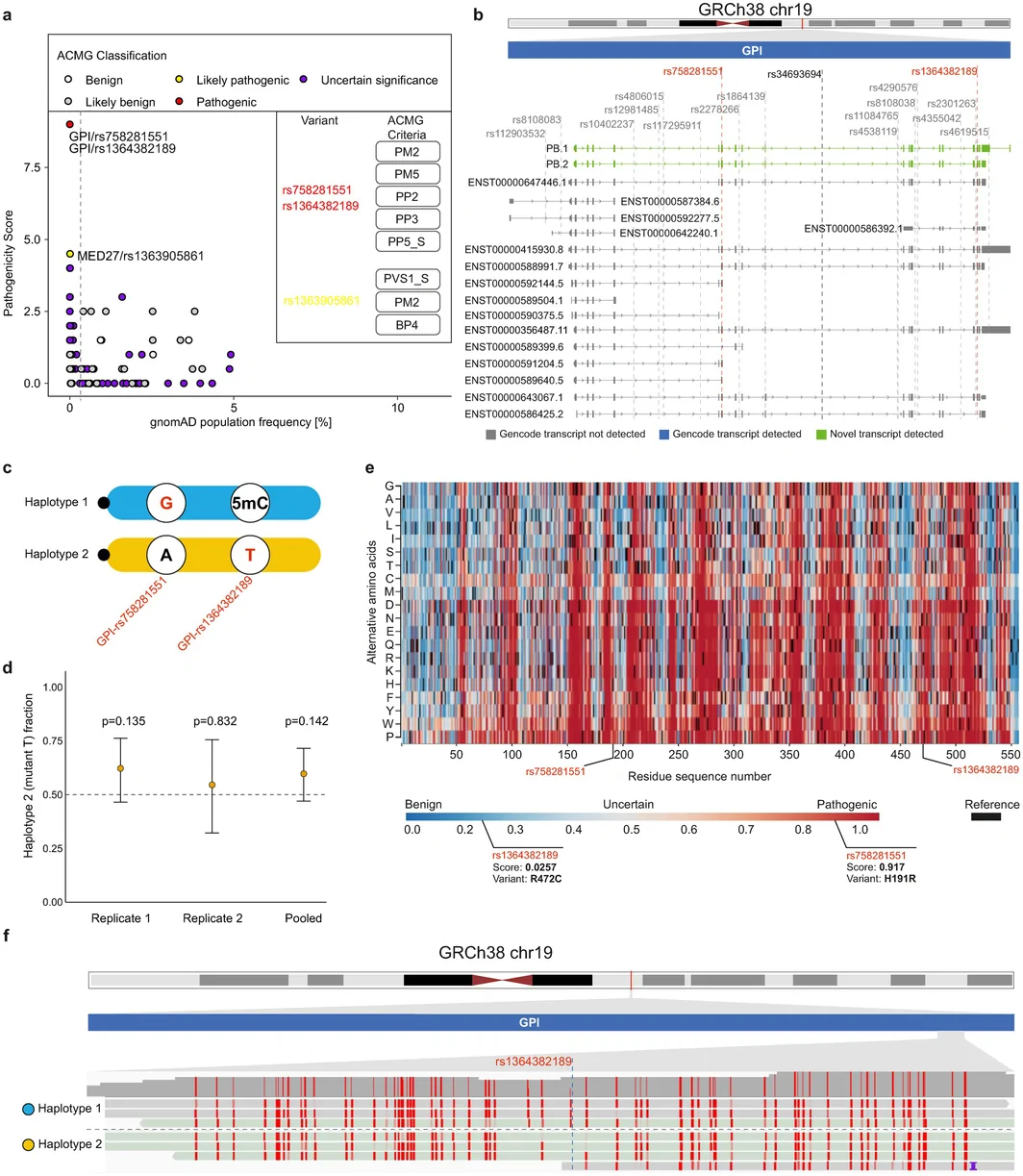
Multiomic view of a GPI‐deficient patient. (a) WGS coding variant classification according to ACMG criteria. (b) Transcriptome assembly of a GPI‐deficient patient with polymorphic and disease‐linked variant positions marked. (c) GPI gene haplotype reconstruction. (d) Relative expression of the GPI gene according to haplotype. (e) AlphaMissense prediction of the impact of pathogenic variants on protein function. (f) Whole Genome Methylation results for GPI gene haplotypes.

Until now, whole exome sequencing (WES) results have been reported for only a few patients with GPI deficiency, and only a single case report with WGS was published [6]. We integrated short‐read Illumina WGS with high‐fidelity (HiFi) long‐read sequencing. We identified two heterozygous missense variants in the GPI gene (NM_000175.5): a c.572A>G variant (rs758281551, p.His191Arg) and a c.1414C>T variant (rs1364382189, p.Arg472Cys) (Figure 1a and Table S1). Both variants are extremely rare in the European population (gnomAD frequencies < 0.0001).

Long‐read data allowed direct molecular phasing, placing these variants in trans configuration (Figure 1c). Each variant marks a distinct haplotype: Haplotype 1 carries p.His191Arg, while Haplotype 2 carries p.Arg472Cys. This confirmed the patient as a compound heterozygote, with no wild‐type GPI allele detected. We also identified 15 non‐coding variants within the GPI locus (Figure 1b); deep‐learning predictions (SpliceAI) indicated no disruption to canonical splicing motifs above the standard reporting threshold (SpliceAI delta‐score 0.5).

### 2.2 Allele‐Resolved Transcript Counts Show a Directionally Consistent but Non‐Significant Trend Toward Higher Haplotype 2 Expression

To assess whether the identified variants were associated with a difference in transcriptional output, we performed full‐length isoform sequencing (Iso‐Seq) on whole‐blood RNA in two independent technical replicates (separate SMRT Cells). We assembled the GPI transcriptome and identified two novel minor isoforms (PB.1 and PB.2) in addition to the canonical transcript (Figure 1b); these isoforms are described below as a descriptive finding and are not incorporated into our mechanistic hypothesis.

We quantified haplotype‐resolved read counts at the phased heterozygous positions. Across both replicates, Haplotype 2 (carrying p.Arg472Cys) showed a directionally consistent trend toward higher representation than Haplotype 1; however, the magnitude was modest and one replicate was close to the expected 50:50 read split (Figure 1d). Per‐replicate allele read counts, allelic fractions, and 95% confidence intervals are reported in Table 1.

**TABLE 1:** Allele‐specific GPI transcript counts per Iso‐Seq replicate and pooled. Haplotype 1 carries the wild‐type C at c.1414 (p.His191Arg); Haplotype 2 carries the mutant T (p.Arg472Cys). Fractions are the Haplotype 2 proportion; 95% CIs are exact (Clopper–Pearson); *p*‐values are two‐sided exact binomial tests against an expected 0.5 fraction.

Sample	Hap1_C	Hap2_T	Total	Hap2_fraction	CI95_low	CI95_high	*p*
Replicate_1	17	28	45	0.6222	0.4654	0.7623	0.135
Replicate_2	10	12	22	0.5455	0.3221	0.7561	0.832
Pooled	27	40	67	0.597	0.47	0.7151	0.142

At the read level, the allelic imbalance did not reach statistical significance in both replicates, and the two replicates were concordant only in the direction, not the magnitude, of the effect. We therefore describe this as a non‐significant trend toward allele‐specific expression (ASE) favoring Haplotype 2, rather than as an established ASE effect.

Isoform‐resolved allelic quantification (i.e., ASE restricted to individual isoforms) was underpowered: the number of full‐length reads that unambiguously assigned both a haplotype and a specific isoform was too low to support a formal test, and no isoform‐level allelic conclusion was drawn.

### 2.3 The c.1414C>T Variant Disrupts a Local CpG Dinucleotide

We next examined whether the c.1414C>T variant altered the local methylation landscape. Leveraging the polymerase kinetics of PacBio HiFi sequencing, we obtained native 5‐methylcytosine (5mC) calls across the GPI locus without bisulfite conversion (Figure 1f). The wild‐type cytosine at position c.1414, retained on Haplotype 1, lies within a CpG dinucleotide context. In the small number of HiFi Haplotype‐1/wild‐type reads spanning this position (*n* = 7 reads), the site was called methylated in all reads with the C allele.

The pathogenic c.1414C>T transition on Haplotype 2 removes this cytosine and therefore abolishes the CpG dinucleotide (Figure 1f). Because the CpG is destroyed on Haplotype 2, this allele cannot carry a 5mC mark at this position by definition. We emphasize the very limited read support underlying this observation (*n* = 3 informative reads) and therefore present it as an observational finding. The co‐occurrence of CpG disruption on Haplotype 2 and the directional ASE trend toward Haplotype 2 is noted as a correlation in this patient; it does not, on its own, establish that loss of methylation drives transcription.

### 2.4 AlphaMissense In Silico Predictions for the Two Variants

As an in silico annotation, we retrieved AlphaMissense pathogenicity predictions for the two variants (Figure 1e). p.His191Arg (Haplotype 1) received a high score (0.914), while p.Arg472Cys (Haplotype 2) received a lower score (0.257). AlphaMissense is a computational predictor and is not functional evidence; we include it only as annotation. The relatively low score for p.Arg472Cys is consistent with the possibility that its pathogenicity operates through protein instability rather than through disruption of the catalytic site—an effect not necessarily captured by structure‐based pathogenicity predictors—but this interpretation is a caveat about predictor limitations, not positive mechanistic support.

### 2.5 The Haplotype 2 Variant Encodes a Previously Reported Unstable Protein

Prior biochemical characterization of p.Arg472Cys established it as a thermally unstable variant prone to rapid degradation [7]. In the present study, we did not measure GPI protein abundance, enzymatic activity, or protein stability in this patient; the instability of this variant is taken from the published literature and from computational prediction, not from direct measurement in this case. Consequently, any model that couples the observed transcript‐level trend to protein turnover remains hypothetical in this patient.

## 3 Discussion

The molecular diagnosis of rare enzymopathies has traditionally been framed around the structural damage a variant inflicts on the encoded protein. Integrative, multi‐layered characterization of the locus offers a complementary perspective. Applying long‐read phasing, native methylation calling, and full‐length transcriptomics to a single patient with GPI deficiency, we identified a candidate “dual‐effect” variant, c.1414C>T (p.Arg472Cys) that simultaneously alters the coding sequence and abolishes a local CpG dinucleotide.

Three descriptive conclusions are robustly supported by the data. First, haplotype‐resolved sequencing establishes that the proband is a compound heterozygote carrying both GPI variants in trans, with no wild‐type allele. Second, full‐length transcriptomics resolves the GPI transcriptome at this locus and reveals two previously undescribed minor isoforms. Third, native methylation calling shows that c.1414C>T destroys a CpG dinucleotide whose cytosine is methylated on the wild‐type background in the reads observed.

Our observations nonetheless motivate a testable model. If c.1414C>T does modestly de‐repress its own allele—for example through loss of a local methylation mark—and if the encoded protein is unstable as previously reported [7], then a compensatory increase in transcription could partially offset rapid protein turnover in nucleated erythroid precursors. Such compensation would necessarily be lost upon enucleation and the cessation of transcription, potentially contributing to a cell‐age‐dependent decline in GPI activity of the kind proposed in earlier kinetic studies of hemolytic anaemia [8].

Testing this model would require, at minimum, four lines of evidence: allele‐specific quantification of GPI transcripts at adequate read depth with formal control for mapping bias; a quantitative, statistically powered, haplotype‐resolved comparison of methylation at this and neighbouring CpGs, ideally across cell fractions; a direct experimental test of whether this CpG carries any regulatory activity, such as a reporter assay or targeted (de)methylation; and direct measurement of GPI protein level, stability, and enzymatic activity, ideally comparing reticulocyte‐enriched with mature‐erythrocyte fractions.

Although the p.His191Arg variant has been associated with neurological features in prior reports [9, 10], our proband presented with an exclusively haematological phenotype. One speculative explanation is that residual enzymatic flux from the trans allele suffices to protect neural tissue, which—unlike the mature erythrocyte—retains nuclear capacity for continuous transcription. In a single patient this remains conjecture, and we offer it only as a direction for further study.

Finally, the case illustrates the practical value of long‐read multi‐omics in the diagnostic setting. PacBio HiFi sequencing permitted simultaneous phasing of variants and direct detection of native methylation without bisulfite conversion, yielding an integrated view of a locus that short‐read exome or genome workflows would not fully resolve. The added value demonstrated here, however, is the generation of mechanistic hypotheses about a variant of uncertain significance—not the establishment of a mechanism.

In conclusion, this molecular case study illustrates how integrative long‐read multi‐omics can generate specific, testable hypotheses about how a coding variant might additionally influence its own regulatory context. We do not claim to have established a new class of pathogenic mechanism; rather, we describe a candidate dual‐effect variant and define the functional experiments required to test whether transcriptional output, epigenetic state, and protein stability interact to shape disease in GPI deficiency. As long‐read multi‐omics becomes more accessible, systematic evaluation of such candidate dual‐effect variants across additional patients and loci will be needed to determine whether they represent a recurrent phenomenon.

## 4 Limitations

The observations reported here are correlative, derive from *n* = 1, and are not accompanied by functional or protein‐level validation; we therefore present our interpretation as a hypothesis to be tested rather than as an established mechanism. Several limitations bound what may be inferred from these findings. The data do not establish that methylation at this CpG regulates GPI transcription, nor that loss of the methylation mark causally drives allele‐specific expression. Allele‐resolved read counts show only a directionally consistent trend that does not reach statistical significance, and the methylation observation rests on a small number of reads. We did not measure GPI protein abundance, protein stability, or enzymatic activity in this patient. Any causal reading of the variant as a cis‐regulatory element governing expression at this locus would therefore outrun the present evidence.

## 5 Methods

### 5.1 Short Read Illumina Whole Genome Sequencing (WGS) and Data Analysis

Whole blood from 47‐year‐old woman who presented with hemolytic anaemia was collected in an EDTA tube. High‐quality DNA was isolated with QIAGEN blood&tissue kit. Next Generation Sequencing (NGS) library was built starting from 500 ng of DNA with Illumina DNA Prep kit and sequenced on Illumina NovaSeq 6000 system with 2 × 151bp paired‐end mode, resulting in > 30× genome coverage.

The sequencing data underwent quality control with FastQC software and were quality trimmed with AdapterRemoval. Next, reads were aligned to the human reference genome ver. GRCh38 with bwa mem. SNVs and short indels were called using the Genome Analysis Tool Kit (v4.1.1.9, https://gatk.broadinstitute.org/) and best practices for germline calling (hard‐filtering: SNPs—QD < 2, FS > 60, MQ < 40, MQRankSum < −12.5, ReadPosRankSum < −8, SOR > 3; indels—QD < 2, FS > 200, ReadPosRankSum < −20, SOR > 10), and annotated using Ensembl Variant Effect Predictor (v97, https://github.com/Ensembl/ensembl‐vep).

### 5.2 Long‐Read 5‐Base PacBio HiFi WGS and Data Analysis

High molecular weight DNA was fragmented using Covaris g‐TUBES to an average fragment length of 14.1 kb. A whole‐genome library was then prepared from 1.48 μg of this DNA using the SMRTbell Express Template Prep Kit 3.0 and the Sequel II Binding Kit 3.2. WGS run was set up with Sequel II Sequencing kit 2.0 at 80 pM on‐plate loading concentration in two 8 M SMRTcells on the Sequel IIe sequencer. The resultant data were processed with SMRT Link ver. 11.0. The HiFi reads were generated by a consensus of at least nine raw reads, minimum CCS predicted accuracy QV > 20. The HiFi data were aligned to the human reference genome ver. GRCh38. The WGS and 5‐mC data alignment results were manually inspected in the Integrative Genomic Viewer software at selected positions identified as rare variants by the Illumina short‐read sequencing.

### 5.3 RNA Isolation and Long‐Read PacBio Iso‐Seq Library Preparation and Transcriptome Assembly

Total RNA was isolated for GPI patient and healthy control from blood collected to the PAXgene tubes, with the use of PAXgene Blood RNA Kit (PreAnalityX, Qiagen), including on‐column DNase treatment. RNA quantity and quality were assessed using a Qubit fluorometer (Thermo Fisher) and Bioanalyzer 2100 (Agilent Technologies), respectively, with sample exhibiting RNA integrity numbers (RIN) of 8.4. PacBio Iso‐Seq unbarcoded libraries were prepared from 500 ng of total RNA using the NEBNext Single Cell/Low Input cDNA Synthesis and Amplification Module (New England Biolabs) with the Iso‐Seq Express oligo kit (Pacific Biosciences) for cDNA synthesis, and then library preparation was completed using SMRTbell Express Template Prep Kit 3.0 and SMRTbell Library Binding Kit 3.1 (Pacific Biosciences) following the manufacturer's protocol. Libraries quantified via Qubit and Bioanalyzer HS RNA screen tape were sequenced with Sequel II Sequencing kit 2.0 on a Sequel IIe system (Pacific Biosciences) at 80 pM on‐plate loading concentration with 24‐h movie times. Two technical replicates were sequenced across independent SMRT Cells. The resultant data were processed with SMRT Link ver. 11.0. The HiFi reads were generated by a consensus of at least nine raw reads.

### 5.4 Allele‐Specific Expression Analysis

Allele‐specific expression was assessed directly from the standard Iso‐Seq full‐length transcript reads, without read haplotagging. For each Iso‐Seq replicate, full‐length reads spanning the phased heterozygous marker position c.1414 (C on Haplotype 1, T on Haplotype 2) were inspected, and each read was assigned to Haplotype 1 or Haplotype 2 according to the base (C or T) it carried at that position; reads that did not span c.1414 or in which the base could not be unambiguously determined were excluded. Allele‐specific read counts were tabulated per replicate and pooled using an in‐house script. Deviation from the expected 50:50 allelic ratio was tested with a two‐sided exact binomial test at *α* = 0.05, reported for each replicate and for the pooled data; 95% confidence intervals on the Haplotype 2 allelic fraction were computed using the Clopper–Pearson (exact) method. Because the two replicates were technical (independent SMRT Cells from the same RNA sample), *p*‐values were reported unadjusted and interpreted conservatively in light of the per‐replicate concordance. We note that this simple read‐counting approach does not formally correct for reference‐allele mapping bias, a known confounder of allele‐specific quantification from aligned reads.

## Author Contributions

**Jarosław Czyż:** conceptualization, investigation, writing – original draft, writing – review and editing, validation, resources, supervision, project administration. **Luiza Handschuh:** resources, supervision, writing – review and editing. **Marek Figlerowicz:** resources, supervision, validation, funding acquisition. **Małgorzata Marcinkowska‐Swojak:** methodology, formal analysis, investigation. **Ireneusz Stolarek:** conceptualization, investigation, funding acquisition, writing – original draft, methodology, validation, visualization, writing – review and editing, formal analysis, project administration, data curation, supervision. **Magdalena Rakoczy:** investigation, methodology, formal analysis. **Joanna Delimata‐Raczek:** investigation, validation, writing – original draft. **Klaudia Sikora:** investigation, methodology, data curation. **Natalia Koralewska:** conceptualization, methodology, project administration, writing – review and editing, funding acquisition.

## Funding

This work was supported by the National Information Processing Institute, POIR.04.02.00‐00‐D017/20.

## Conflicts of Interest

The authors declare no conflicts of interest.

## Supporting information


**Table S1:** VarSome assigned pathogenicity classification and variant annotations.

## Data Availability

The data that support the findings of this study are available on request from the corresponding author. The data are not publicly available due to privacy or ethical restrictions.
